# Caregiving in the U.S. 2020: What Does the Latest Edition of This Survey Tell Us About Their Contributions and Needs?

**DOI:** 10.1093/geroni/igaa057.2371

**Published:** 2020-12-16

**Authors:** Gabriela Prudencio, Heather Young

## Abstract

Family and friends comprise the most basic unit of any society. For individuals who take on the responsibility of caring for another person through sickness or disability, it can often be challenging to see beyond the individual experience. Yet in the aggregate, family caregivers—whether they be families of kin or families of choice—are woven into the fabric of America’s health, social, economic, and long-term services and supports (LTSS) systems. As the country continues to age, the need to support caregivers as the cornerstone of society will only become more important. A national profile of family caregivers first emerged from the 1997 Caregiving in the U.S. study. Related studies were conducted in 2004, 2009, and 2015 by the NAC in collaboration with AARP. Caregiving in the U.S. 2020 presents a portrait of unpaid family caregivers today. A nationally representative survey (n=1,499), it replicates the methodology used in 2015. Therefore, during this symposium, AARP and NAC will present trend data from 2015 in comparison to 2020, and explore key subgroup differences. The presentation will cover prevalence, demographic characteristics, intensity and duration of care, the well-being of caregivers, the financial impact of family caregiving on caregivers themselves, and the degree to which technology supports caregivers today.

